# Multimorbidity: Epidemiology and Models of Care

**DOI:** 10.1155/2016/7029027

**Published:** 2016-03-27

**Authors:** A. Marengoni, R. J. F. Melis, A. Prados Torres, G. Onder

**Affiliations:** ^1^Department of Clinical and Experimental Sciences, University of Brescia, 25123 Brescia, Italy; ^2^Radboud University Nijmegen Medical Centre, 6500 HB Nijmegen, Netherlands; ^3^IIS Aragon, Aragon Health Sciences Institute (IACS), 50009 Zaragoza, Spain; ^4^Department of Geriatrics, Catholic University of Sacred Heart, 00168 Rome, Italy

Due to the aging of the population, the prevalence of chronic diseases is progressively increasing and most older adults experience the cooccurrence of multiple diseases, a condition known as multimorbidity. It has been estimated that 60% of persons aged 65 years or older are affected by multimorbidity, the reason why the condition is sometimes referred to as the “most common chronic disease” [1]. The appearance of clusters and patterns of patients and diseases in different context and populations group has been also demonstrated [2, 3]. Advanced age, female gender, low socioeconomic status, and education are among the main risk factors for the development of multimorbidity. This suggests, for example, that early life learned risk behaviours may affect the development of this condition [2]. Compared to those with single conditions, persons with multimorbidity are more likely to experience negative health outcomes, including mortality, hospitalization, and functional and cognitive decline, leading ultimately to poorer quality of life and increased care costs. Persons with multimorbidity have the most complex health needs but, due to the current disease-oriented approach in healthcare, they face highly fragmented care that leads to incomplete, inefficient, ineffective, and even potentially harmful interventions [4].

In this special issue, investigators reported studies into the subject from all over the world (i.e., Canada, India, Panama, Portugal, Netherlands, and UK). They contributed to increasing the knowledge on multimorbidity by focusing on clustering of chronic diseases and methods to evaluate multimorbidity and its impact on clinical outcomes, including functional status, quality of life, compliance to physical activity, depression, and cognitive impairment. Sanghamitra Pati and colleagues described and validated a new tool for multimorbidity assessment in India. Although it is known that low and middle income countries with socioeconomic development and westernization of lifestyle are no longer “immune” to multimorbidity, multimorbidity is still underexplored in these countries. This study definitively contributed to estimating the magnitude and impact of multimorbidity in primary care practice populations in developing countries. Joanna Collerton and colleagues examined the extent and complexity of the morbidity burden in the Newcastle 85+ Study, a population-based cohort study. The authors used cluster analysis to identify patterns of diseases within multimorbidity and to compare clusters on medication and healthcare use. A cluster approach was used also by Sarah Dörenkamp and colleagues. Their objective was to identify clusters of multimorbidity associated with physical activity, using data from the Dutch cohort study SMILE. They evidenced that the lowest rate of physical activity and guideline compliance was reported in patients with heart disease, respiratory disease, and diabetes mellitus. Several potential uses of a cluster medicine approach deserve to be highlighted: (1) New research hypotheses on possible shared pathological pathway for clusters of specific diseases can be developed. (2) Prevention can be implemented. (3) Groups of people at high risk of adverse outcomes can be identified. (4) Prevalence of use of potentially inappropriate medication or adverse drug reactions could be higher in different clusters. (5) Clinical trials could be carried out in groups of the elderly affected by specific clusters of diseases. (6) Treatment can be better tailored to the individual person because it enables actively evaluating the presence of and dealing with comorbidities known to cooccur. (7) Finally, the severity of a disease can be approximated by its connections with other diseases for patients with the same number of diagnoses [3, 5].

Filipe Prazeres and colleagues described the translation of the European General Practice Research Network Multimorbidity definition according to Portuguese cultural and linguistic features. The definition of multimorbidity is now available in a new language, Portuguese. The operationalization of the definition and its availability in the local language will raise Portuguese GPs' awareness about multimorbidity and allow future national and international research. Villarreal and colleagues reported first data on the association of multimorbidity with the cooccurrence of cognitive impairment and depression alone in older persons living in Panama. In the older population, depression is frequent and it also commonly coexists with other chronic medical conditions. On one hand, chronic diseases increase the risk of depression due to the presence of disability, pain, and polypharmacy in multimorbid persons. On the other hand, depression can negatively affect adherence to medications and to a healthy lifestyle that are needed to prevent other clinical conditions. This points at the complexities underlying disease cooccurrence and the mechanism of reciprocity, which is a phenomenon that is perhaps understudied in medicine to understand the relationships between determinants and outcomes. Finally, Aline Ramond-Roquin and colleagues evaluated the association between different multimorbidity measures and physical quality of life. Studies aiming to quantify the impact of multimorbidity on quality of life showed wide heterogeneity in terms of the intensity of this association. It has been suggested that the lack of a uniform way to measure multimorbidity may explain a significant part of this variability. The length of the list of candidate conditions considered has a great impact on the estimations of physical health-related quality of life. The selection of different methods to measure multimorbidity is critical in determining both prevalence of multimorbidity and its association with the outcome of interest. The simple count of diseases has both advantages and disadvantages. A relevant advantage of this approach is that it expresses multimorbidity in an additive form, and it conveniently differentiates people at each level of morbidity. Second, each individual disease contributes to the disease count, avoiding problems of insufficient statistical power, especially if rare diseases are evaluated. On the contrary, one of the most reported disadvantages is that all diseases are scored equally, independently of their severity.

Despite the increasing interest of the researchers in this field, there is still a remarkable gap between the harmful impact of multimorbidity at the individual and societal level and the amount of scientific and clinical research devoted to this topic. Contributions to this special issue filled some gaps in the field providing useful tools to measure multimorbidity and data exploring the prevalence, type, and impact of the presence of multiple cooccurring diseases.

*A. Marengoni* *R. J. F. Melis* *A. Prados Torres* *G. Onder*
